# Ex Situ Raman Mapping of LiMn_2_O_4_ Electrodes Cycled in Lithium-Ion Batteries

**DOI:** 10.1021/acsomega.4c01480

**Published:** 2024-07-01

**Authors:** Dominika A. Buchberger, Bartosz Hamankiewicz, Monika Michalska, Alicja Głaszczka, Andrzej Czerwinski

**Affiliations:** † Faculty of Chemistry, University of Warsaw, Pasteura 1, 02093 Warsaw, Poland; ‡ Faculty of Materials Science and Technology, VSB-Technical University of Ostrava, 17. listopadu 2172/15, 708 00 Ostrava-Poruba, Czech Republic

## Abstract

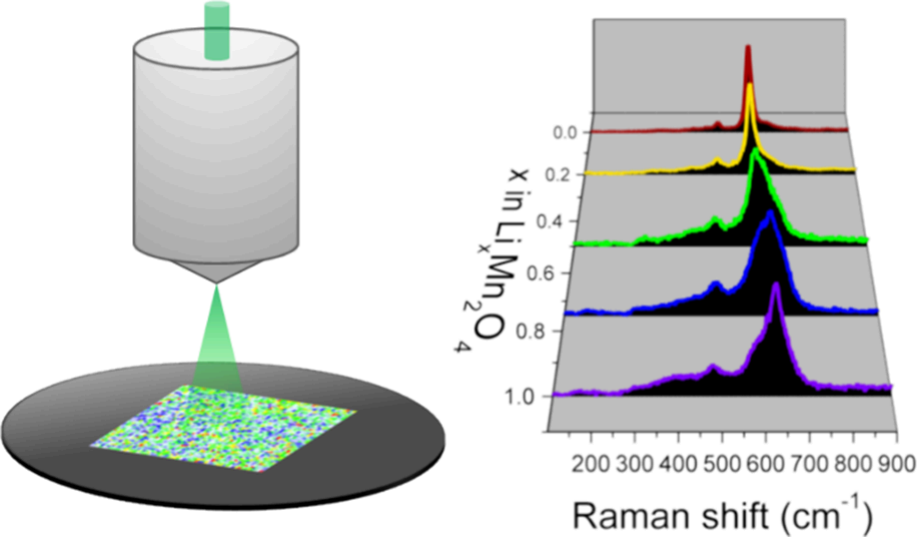

In this study, we focus on the large-scale ex situ Raman mapping of LiMn_2_O_4_ (LMO) electrodes maintained at varying states of charge. A comprehensive statistical analysis has been conducted at an area of ca. 3660 μm^2^ on more than 3100 collected spectra for each LMO electrode sample. High-definition ex situ Raman maps provide profound insight into the lithiation process, offering an additional perspective on the mechanism of LMO intercalation. These maps clearly depict the coexistence of two phases, with evident phase transitions and state-of-charge gradients. The set of spectra with various state-of-charge has been successfully deconvoluted taking into account the two-phase character of the ongoing reaction. In addition, we performed the study on the samples operated for 50 cycles at the high C-rates and tracked their delithiation state and impurity formation. This technique serves as a complementary visualization and analytical tool alongside other bulk-type methods employed in battery diagnostics. Importantly, this ex situ Raman mapping approach is applicable to any electrode material exhibiting a Raman response.

## 1 Introduction

A spinel lithium manganese oxide (LiMn_2_O_4_) material is one of the cathode structures applied in secondary lithium-ion batteries. Several important advantages make it a suitable alternative to other currently researched materials, such as layered transition metal oxides. These benefits comprise its low cost, easy and nontoxic preparation, high discharge potential (4 V vs Li^+^/Li), practical capacity of 120 mAh g^–1^, high-energy density, and low self-discharge.^1−3^ However, LiMn_2_O_4_ (LMO) experiences a capacity fading during cycling, which restrains its applicability in commercial lithium-ion batteries.^4,5^ To understand this undesirable trend, structural investigations over cycling have to be carried out. In recent years, many bulk- and surface-type analyses such as in situ and ex situ techniques were demonstrated.^6−13^ Raman spectroscopy stands out as a significant structural technique, primarily owing to its remarkable attributes such as high sensitivity, noninvasive nature, and nondestructive characteristics. More recently, the combination of optical microscopy and Raman spectroscopy has provided an additional advantage of efficiently scanning large sample areas. This renders it an ideal tool for examining structural changes in electrode materials.

One of the first Raman spectra interpretations of the LMO spinel structure can be found in previous works of Julien et al.^14−16^ Since then, the history of in situ and ex situ Raman analyses on the Li^+^ intercalation process in LMO microcrystals has continued over 20 years and resulted in several important reports.^17−28^ The first in situ measurement of Li^+^ electro-insertion into a Pt/λ-MnO_2_ electrode in a 0.1 M LiCl aqueous solution was presented in 1998 and showed that in situ recorded spectra were very similar to their ex situ data.^17^ One of the most important Raman studies on the LiMn_2_O_4_ material was introduced by Ammundsen et al. in 1999.^18^ Their theoretical calculations on expected Raman-active modes in LMO spinel structures coupled with subsequent experimental evidence validating the calculated Raman shift positions render this research one of the most significant and valuable contributions in the field. They performed additional ex situ Raman measurements on two electrochemically delithiated and half-lithiated materials: λ-MnO_2_ and Li_0.5_Mn_2_O_4_. The theoretical calculation model was further developed by M.M. Sinha and H.C. Gupta in 2002 and added a new interpretation to the available experimental data.^29^

The first in situ Raman research on the Li-ion cell prepared in an Ar atmosphere was performed by W. Huang and R. Frech.^19^ A two-electrode cell setup composed of the LMO cathode and lithium metal was assembled using a 1 M LiClO_4_ solution in 1:1 wt EC:DMC. The cell was measured in both directions (over charge and discharge processes). The authors stated that during the in situ Raman study of the LMO electrode, the spectral changes indicated that a single-phase reaction is followed by a two-phase reaction and then by another single-phase reaction as Li^+^ ions are deintercalated from the stoichiometric LiMn_2_O_4_. When the spinel LMO structure is further intercalated with Li^+^ forming the Li-rich phase, the in situ Raman spectral changes are consistent with a two-phase reaction between cubic LiMn_2_O_4_ and tetragonal Li_2_Mn_2_O_4_. In situ Raman measurements were continued between 2001 and 2005. Raman spectra of isolated single particles of LMO embedded in Au foils were recorded in situ in 1 M LiPF_6_ in 2:1 wt EC:DMC using a Raman microscope by D. Scherson’s group.^20^ Authors correlated their in situ spectroscopic data of a single LMO particle directly with its electrical state. Their other work was based on simultaneous Raman spectra and CV data acquisition from the LMO single particle microelectrode immersed in a 1 M LiClO_4_ 1:1 vol EC:DEC solution.^21^ Statistical analyses were conducted on the spectra within the range of 15% < SOD < 45% (SOD—state of discharge). The results were consistent with the coexistence of two distinct phases of lithiated metal oxide and well-agreed with in situ XRD measurements reported previously.^30−32^ The first in situ Raman spectra collection on Li_1–*x*_Mn_2_O_4_ thin films produced by electrostatic spray deposition was recorded during a CV scan in 1 M LiClO_4_ in PC:EC solutions.^22^ Authors suggested that each value of *x* in Li_1–*x*_Mn_2_O_4_ possesses its own electronic band structure.

In 2005, a very interesting and thorough in situ Raman study was performed on a LiMn_2_O_4_ single-crystal microelectrode recorded as a function of potential vs Li in a 1 M LiPF_6_ 1:1 vol EC:DMC solution.^24^ Based on this study, it was clearly evident that two well-defined steps were involved in the charge process of the LMO material and were in agreement with the voltammetric characteristics The corresponding SOD (state of discharge) vs *E* plots, which were obtained by integration of the linear scan voltammogram, were very similar in shape.

A couple of the most recent ex situ Raman studies cover the study of commercial LMO electrodes at their charged and discharged states^25^ and the correlation between the electrochemical data and structural responses of LMO electrodes charged to high anodic potentials of 4.3 to 5.1 V vs Li/Li^+^.^26^ Lately, the Raman mapping was also employed to study the LMO grains in two lithiated states *x* = 0.1 and 0.4 before and after aging (discharged at 1 and 16 C, respectively) of the commercial LMO electrodes.^27^ The authors stated that cycling leads to (1) the formation of the Mn_3_O_4_ phase with its further dissolution in the electrolyte and (2) qualitative change in the lithiation process in cycled LMO cathodes with significant inhomogeneity of the formed lithiation state. The summary of the state-of-the-art in terms of in situ and ex situ Raman studies on LMO materials is presented in Tables 1 and S1 (extended).

**Table 1:** Literature Data on LiMn_2_O_4_ Were Obtained Using Ex Situ and In Situ Raman Studies

laser used (power)	electrolyte	working electrode	electrochemical cell	novelty	measurement type	year	ref.
Nd:YAG laser at 532 nm (1 mW)	0.1 M LiCl + 0.05 M borate buffer aqueous solution	thin layer electrode Pt/λ-MnO_2_	three-electrode: aqueous	the first in situ Raman study of electrochemical Li insertion in the MnO_2_ spinel structure in aqueous solution.	in situ and ex situ	1998	(17)
Nd:YAG laser at 532 nm (unknown)	0.01 M LiCl aqueous solution	thin film electrode Pt/LMO & further Pt/λ-MnO_2_	two-electrode: aqueous	theoretical calculation of Raman active phonon positions in LiMn_2_O_4_, Li_0.5_Mn_2_O_4_, and MnO_2_ and experimental evidence.	ex situ	1999	(18)
Ar laser: 514.5 nm line (20 mW)	1 M LiClO_4_ in 1:1 wt EC:DMC	standard electrode slurry onto Al meshes.	two-electrode: split-type cell (Ar)	the first in situ Raman studies of LMO electrodes during lithium intercalation in Ar-sealed Li-ion cell cycled between 4.6 and 2.1 V vs Li metal	in situ	1999	(19)
Nd:YAG laser at 532 nm (∼8 mW)	1 M LiPF_6_ in 2:1 wt EC/DMC	LiMn_2_O_4_ particle electrodes in Au foil	two-electrode: split-type cell (Ar)	this research exploits the capabilities of in situ Raman technique for the acquisition of time-resolved Raman spectra of single particles of LiMn_2_O_4_ embedded in Au foil substrate electrodes as a function of the applied potential.	in situ	2001	(20)
Nd:YAG laser at 532 nm (low)	1 M LiClO_4_ 1:1 vol. EC:DEC	microelectrode Pt/LiMn_2_O_4_ microparticle	two-electrode: microelectrode (Ar)	statistical analyses of the spectra in the range 15% < SOD < 45% showed to be consistent with the coexistence of two distinct phases of lithiated metal oxide and agreed well in situ XRD measurements.	in situ	2003	(21)
Ar laser: 514.5 nm line (unknown)	1 M LiClO_4_ in PC/EC	Li_1–*x*_Mn_2_O_4_ thin films produced by electrostatic spray deposition	two-electrode: microelectrode (Ar)	the first to report in situ Raman measurements of structural changes of an electrostatic spray deposited thin-film (without a binder or conductive agents) of Li_1–*x*_Mn_2_O_4_ during lithium insertion and extraction processes.	in situ and ex situ	2003	(22)
Ar laser: 514.5 nm line (10 Wcm-2)	unknown	unknown (Li_0.5_Mn_2_O_4_ sample)	unknown (electrochemical Li extraction)	the study of the local structure of various lithium manganese oxide stoichiometries using both the classical group factor analysis and a local environment model.	ex situ	2003	(23)
Nd:YAG laser at 532 nm (3 mW)	1 M LiPF_6_ in 1:1 vol EC/DMC	LiMn_2_O_4_ single-crystal microelectrode	two-electrode: microelectrode (Ar)	the contributions of the three crystallographic phases of Li_*x*_Mn_2_O_4_ 0 < *x* < 1 as a function of the amount of Li^+^ in the lattice derived from the optical data were consistent with those extracted from a coulometric analysis of the voltammetric curves.	in situ	2005	(24)
unknown	unknown	LMO electrode from commercial cell	two-electrode: standard commercial	ex situ Raman study on the commercial LMO electrodes at their charged and discharged states.	ex situ	2015	(25)
514 nm laser (∼0.23 mW)	1 M LiPF_6_ in 7:3 wt EMC/EC	LiMn_2_O_4_, CB, and PVdF (80:10:10 by weight) on Al foil (∼20 μm thick)	two-electrode: pouch-type (Li as CE and Celgard separator)	the correlation between the electrochemical data and structural responses of LMO electrodes (commercial LMO material) charged to high anodic potentials of 4.3 to 5.1 V vs Li	ex situ	2017	(26)
488 nm laser (unknown)	unknown	LMO electrode from commercial cylindrical cells	two-electrode: commercial cylindrical	the understanding of delithiation and degradation paths by local CRM measurements of the LMO cathode material with different “state of charge” and “state of health” parameters.	ex situ	2018	(27)

In this study, ex situ Raman mapping is applied to investigate LiMn_2_O_4_ electrodes cycled in lithium-ion cells. The main novelty lies in the ability to analyze a high number of spectra from extensive Raman maps covering a substantial surface area (∼3660 μm^2^) of the electrodes exposed to various potentials vs Li. This study presents the first comprehensive investigation of the state of charge gradient among LMO particles within positive electrodes using Raman spectroscopy. Furthermore, the study was carried out on the samples operated at the high C-rates for 50 cycles, following the delithiation state and the formation of impurities. This ex situ Raman approach combined with microscopic imaging improves the understanding of Li intercalation processes in the real battery. Moreover, this measurement approach holds the potential for application to many other electrode materials in the future.

## 2 Experimental Section

### 2.1 Sol–Gel Synthesis of LiMn_2_O_4_

Lithium manganese oxide (LiMn_2_O_4_) powder was synthesized via the sol–gel method using citric acid (C_6_H_8_O_7_·H_2_O, 99.5%, Sigma-Aldrich) and acetic acid (C_2_H_4_O_2_, 99.5%, Sigma-Aldrich) as main and minor complexing agents. Lithium acetate dihydrate (CH_3_COOLi·2H_2_O, 97%, Fluka) and manganese acetate tetrahydrate ((CH_3_COO)_2_Mn·4H_2_O, 99%, Sigma-Aldrich) were used in a molar ratio of Li:Mn = 1:2. The salts were separately dissolved in deionized water, followed by mixing them together and stirring for a few hours. Later, citric acid was slowly added to the solution, followed by acetic acid addition. The ratio of metal to citric acid was 1:1, and the ratio of citric to acetic acids was 1:0.25, respectively. The solution was thoroughly stirred and slowly evaporated. When the solution became a viscous transparent gel, it was dried for a few hours at 150 °C in the air atmosphere and finally ground in an agate mortar to obtain a fine powder. This xerogel was calcined at 300 °C for 7 h to 700 °C for 5 h under air flow at a rate of 5 °C min^–1^. The as-prepared product will be labeled as LMO.

### 2.2 Structural and Morphological Analysis

A scanning electron microscope (Hitachi S5500, Hitachi High Technologies Corporation, Japan) with an accelerating voltage of 5 kV was used for the LMO sample morphology determination and the estimation of the particle size.

A Siemens D-500 X-ray diffractometer with CuK_α_ radiation (λ = 1.542 Å) was used to investigate the phase composition of the LMO powder. The Match! Three and FullProf software were used to calculate the crystal size (using the (111) plane) and lattice parameters. The diffraction pattern was recorded from 10° to 60° using a 0.002° step size (0.04° min^–1^). The Scherrer equation was used to estimate the crystal size.^33^ VESTA software was applied for creating crystallographic structure schematics using .cif files obtained from the Crystallography Open Database.^34^

The *N*_2_ adsorption/desorption experiments were conducted on a Micromeritics ASAP 2060 apparatus at 77.349 K absolute temperature in the relative pressure range of 0.01–0.995 *p*(*p*^0^)^−1^. Adsorption/desorption isotherm analysis was performed on ASAP 2060 software by calculating the specific surface area using the Brunauer–Emmett–Teller (BET) method and the distribution of pores and their volumes using the Barrett– Joyner–Halenda approach for desorption curves.

### 2.3 Sample Preparation

Electrodes were prepared using a standard laboratory procedure by a doctor blade slurry coating method. The slurry has been formed by mixing 80 wt % LiMn_2_O_4_ spinel, 10 wt % carbon VulcanXC72R, and 10 wt % PVdF binder (predissolved in NMP solution). A 200 μm applicator was used in order to receive a thin film coating with similar cathode loadings. The amount of electrode material on Al foil was about 2.5 mg cm^–2^. The round electrodes of 0.9 cm diameter were cut from the foil and pressed under 20 MPa pressure. Before the cells were assembled, the electrodes were dried at 120 °C in vacuum for 15 h.

### 2.4 Electrochemical Measurements

Electrodes containing LiMn_2_O_4_ nanomaterials were cycled versus lithium in the Swagelok-type cells and either fully charged (4.50 V) or discharged (3.55 V) or kept on partially lithiated states in order to study the intermediate structures. The material was electrochemically tested using current densities of 1 to 30 C. For the Raman study, the electrodes were cycled 3 times at the 1C rate and kept at different potentials, namely, 3.55 (100% DoD), 4.01, 4.055, 4.15, and 4.5 V (100% SoC). Next, cells were disassembled, and electrodes were washed carefully in dimethyl carbonate and allowed to dry in an argon atmosphere before further measurements.

Additionally, a series of LMO electrodes were cycled over 50 cycles at 1 C, 2 C, and 5 C-rates and kept at the charged state (4.5 V) for 1 h [constant current constant voltage (CCCV) method]. Similar to the previous set of samples, cells were disassembled, and electrodes were washed carefully in dimethyl carbonate and allowed to dry in an argon atmosphere before performing further measurements.

All potentials are given relative to the lithium electrode (Li/Li^+^) unless otherwise stated.

### 2.5 Raman Analysis

Raman measurements were performed using a Renishaw inVia confocal Raman microscope and a Nd:YAG laser (wavelength: 532 nm; maximum power: 17 mW). The Lorentz function was used to fit the spectral lines. The LMO electrode maps were measured at the area of ∼3660 μm^2^ with a 1.1 μm step (3136 points). Each spectrum was collected for 40 s. The Raman mapping was applied to this study to increase the measurement statics. The intensity of the laser was decreased to avoid the decomposition of the spinel material (0.1 mW). The spectra deconvolution was performed using OriginPro software and the multipeak fitting method. The position of Raman lines originating from all three phases (LiMn_2_O_4_, Li_0.5_Mn_2_O_4_, and λ-MnO_2_) during the electrochemical reaction was estimated using the theoretical predictions calculated in the work of Ammundsen et al. using atomistic simulations.^18^

## 3 Results

### 3.1 Basic Structural Analysis of LiMn_2_O_4_ Powder

**Figure 1:**
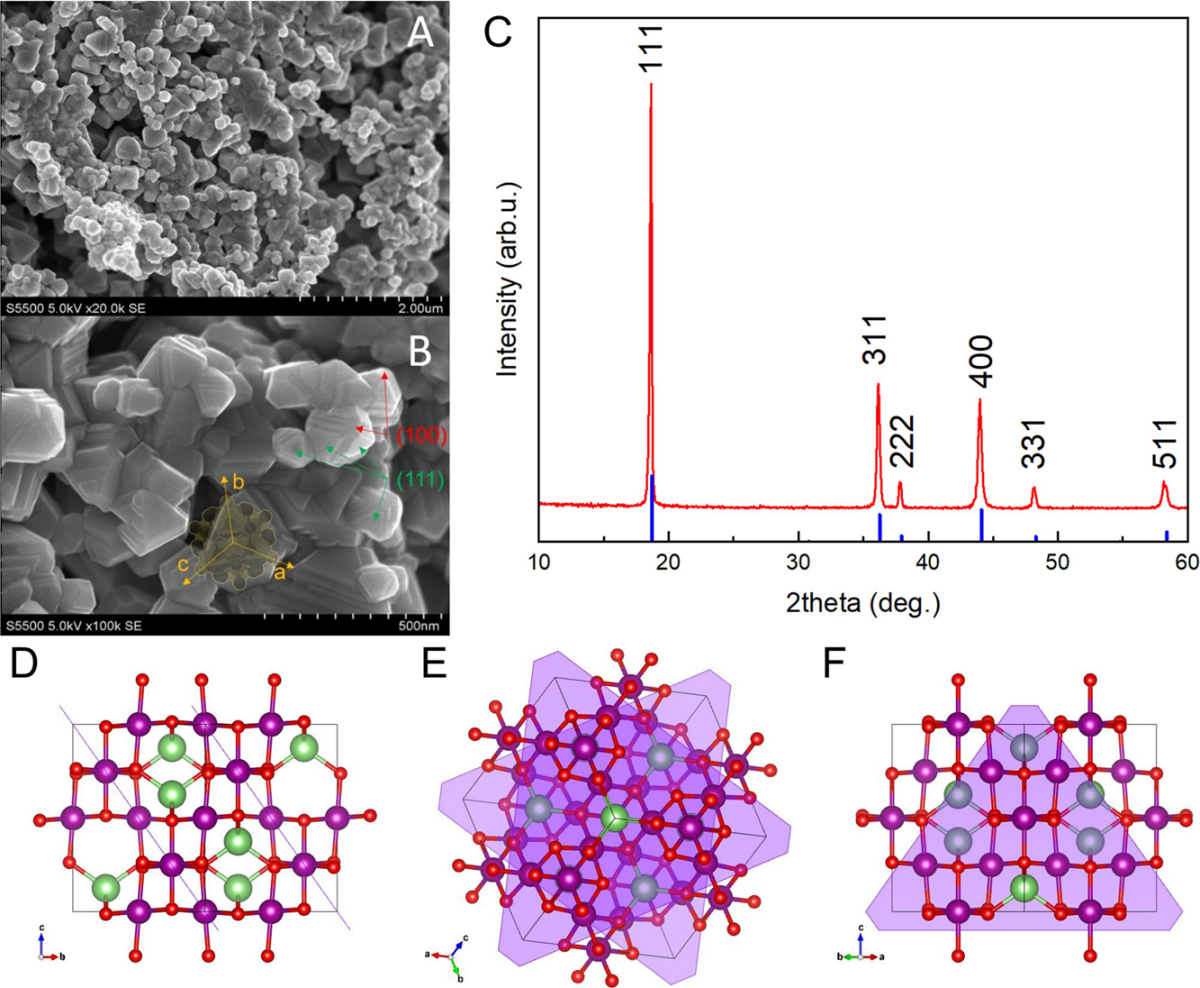
SEM images of the pure LiMn_2_O_4_ material at two magnifications: 20.000× (A) and 100.000× (B). Schematic of the cubic crystal structure (*Fd*3̅*m* space group) is inserted onto the B image to show the selective facet growth in LMO crystals. A characteristic triangular shape of the crystal facets indicates a family of {111} planes. The small areas of the (100) surfaces in truncated cuboctahedrons are also visible. (C) XRD pattern of as-synthesized LiMn_2_O_4_. (D–F) (111) plane presented in different crystallographic directions, where green balls represent Li ions, violet—Mn, and red—oxygens.

SEM images of the LiMn_2_O_4_ powder show well-defined, agglomerated crystals. During the synthesis, they create cuboctahedrons and truncated cuboctahedrons with characteristic triangular facets and plane growth marks close to the edges (Figure 1). These smooth triangular surfaces indicate a selective growth of the direction family ⟨111⟩ (Figure 1B) (all 8 directions) resulting in the well-established (111) facets. Based on theoretical calculations reported previously,^35^ it is predicted that the (111) plane makes the most energetically stable surface facet in the LiMn_2_O_4_ spinel structure. Our experimental results agree well with those estimates that the predominant facet is the (111) plane (followed by two others: (100) and (110) surfaces expected at similar energies). We experimentally observe a cuboctahedral shape with predominant (111) facets, which must possess the lowest surface energies. The truncated cuboctahedrons possess, most probably, additional small (100) surface facets. The representation of the (111) plane in different crystallographic directions is shown in Figure 1D–F, indicating that the Li channels are well exposed for such a morphology. Additionally, SEM imaging reveals a particle size with a distribution of 10 to 400 nm (with an average of about 150 nm).

BET analysis indicates that the specific active area of this LMO powder is 3.18 m^2^/g, whereas the diameters of the dominant pores are about 1.5 and 30 nm. The second pore size value comes from the empty spaces between cuboctahedral grains (Figure 1A, B).

The XRD analysis (Figure 1C) shows that the as-synthesized LMO powder is a single phase of the *Fd*3̅*m* spinel structure (ICDD PDF 35-0782). All characteristic reflexes are indexed in Figure 1C. It is noteworthy that the most intense peak at 18.7° originates from the diffraction of (111) planes, which are represented morphologically through triangular facets on SEM images. The Scherrer formula allows for a calculation of the average crystal size, which is about 75 nm. This fits well with the previous findings of the electron microscopy measurements and suggests the agglomeration of grains into a polycrystal (double-grain and more) particle. Furthermore, the *a* lattice parameter is about 8.246 Å, whereas the cell volume is 560.7 Å, which is consistent with the standard values: *a*_0_ = 8.248 Å and *V*_0_ = 561.1 Å^3^ from the ICDD PDF card.

### 3.2 Electrochemical Testing

**Figure 2:**
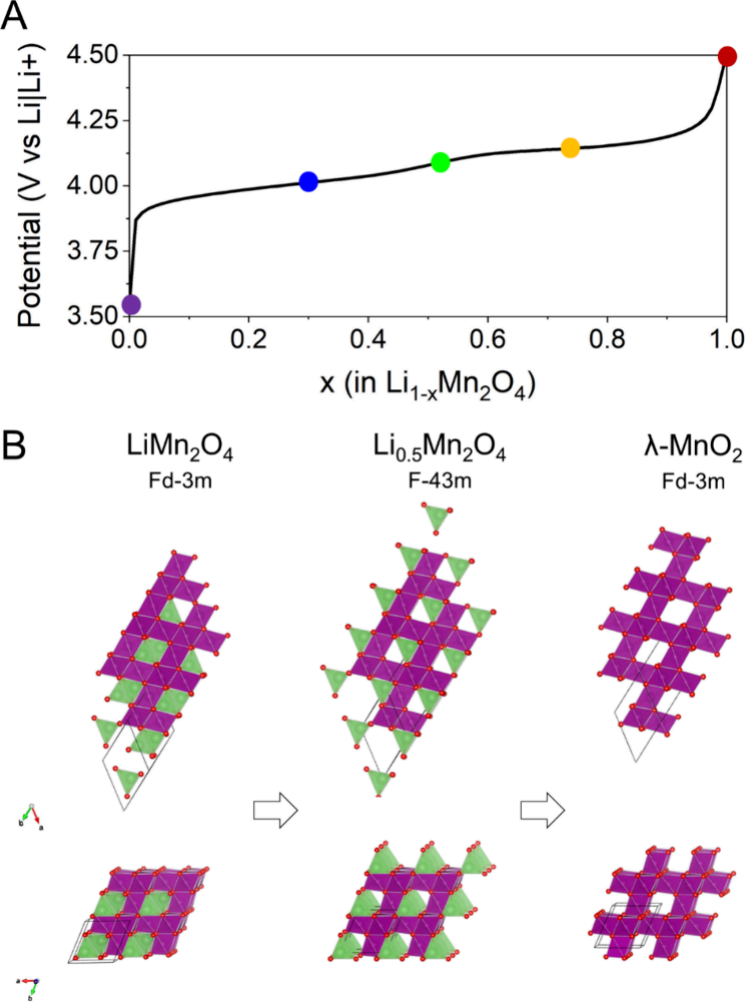
(A) Charging curve showing points at which electrodes were disassembled. (B) Crystal structures showing the evolution of the LiMn_2_O_4_ crystal through Li_0.5_Mn_2_O_4_ and the final λ-MnO_2_ phase.

We conducted a series of chronopotentiometric experiments to explore the electrochemical behavior of LMO materials under different current rates (Figure S1). At a slower current rate of 1 C, the initial specific capacity was found to be 113 mAh g^–1^. To simulate rapid charge and discharge conditions, we measured the electrochemical response at higher current rates of 2, 5, 10, and 30 C and obtained specific capacities of 112, 110, 107, and 99 mAh g^–1^, respectively. After 100 cycles, the materials retained over 95% of their initial capacity. Figure 2A shows the charging curve with indicators showing the potentials at which samples were stabilized and collected for ex situ Raman investigation.

### 3.3 Ex Situ Raman Mapping on Electrodes at Different State-of-Charges

**Figure 3:**
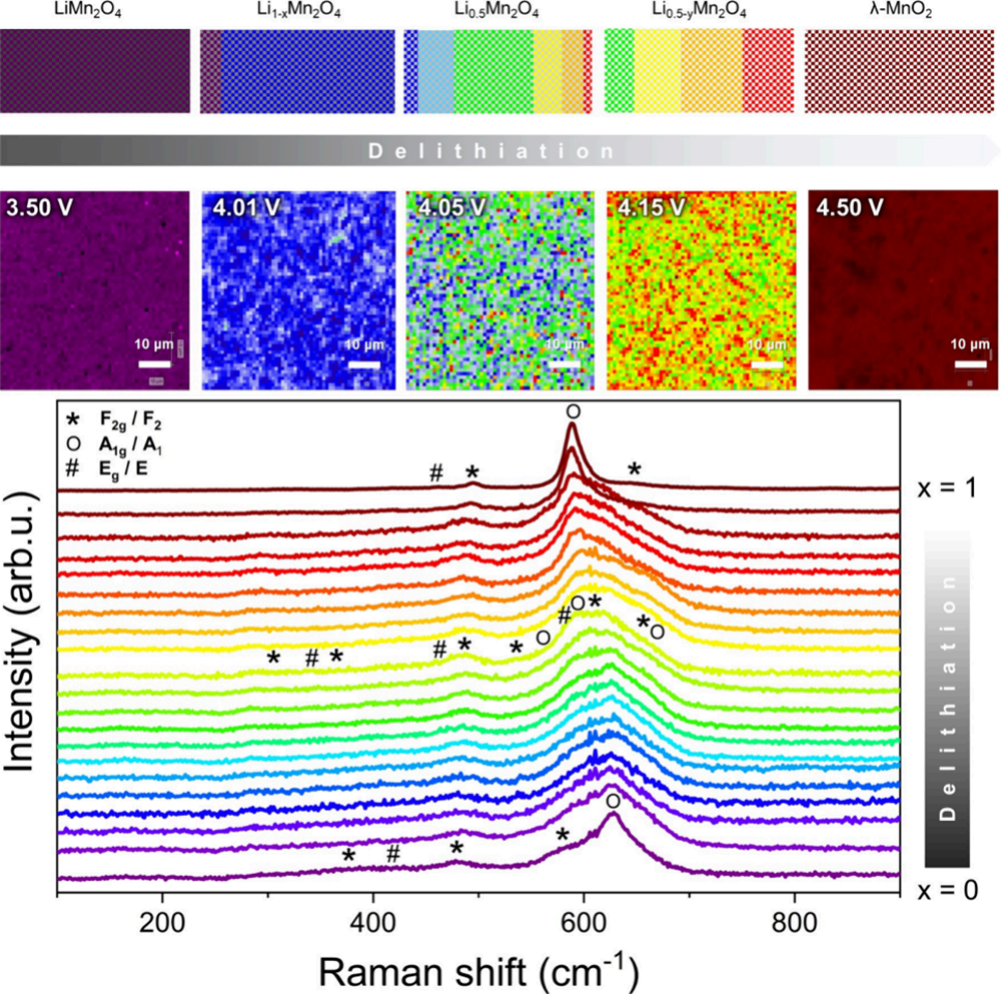
Raman maps and corresponding spectra showing clearly that Li deintercalation is a three-phase process in the Li_1–*x*_Mn_2_O_4_ structure (colors on the maps correspond to the colors of spectra below them). The schematic pictures of the delithiation process are also presented at the top.

A thorough ex situ Raman mapping technique is employed to investigate the changes occurring in the LMO structure during delithiation. After completing 3 cycles, the electrodes are charged to various potentials relative to Li/Li^+^ in order to explore different stages of lithiation. Specifically, we examine the beginning state (3.5 V), the first plateau (4.01 V), the middle state (4.05 V), the second plateau (4.15 V), and the fully charged state (4.5 V) of the electrodes. Figure 2B shows the crystallographic model of the charging process with evolution from the LiMn_2_O_4_ crystal structure through the Li_0.5_Mn_2_O_4_ phase to the final λ-MnO_2_ phase. The electrochemical reaction is described below:
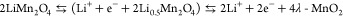


Raman maps perfectly exhibit a stationary state at each potential (Figure 3). Only fully charged and discharged states show very homogeneous spectra over the entire measured area of the electrode. A full discharged state shows that the active electrode material returns to the LiMn_2_O_4_ structure (purple area). A minority of crystallites create the Li-rich Li_1+*z*_Mn_2_O_4_ type of the spinel structure (where *z* is the excess of Li ions)^23^ since a fitting analysis shows that the most intense peak (A_1g_ phonon mode) shifts to a higher frequency region (from 627 cm^–1^ up to 635 cm^–1^) for those few spectra (Figure S2). This indicates a shortening of the Mn–O bond. It is known that in Li-rich LMO (Li_1+*z*_Mn_2_O_4_ compounds), Li ions occupy available 16d octahedral sites and cause a distortion of MnO_6_ octahedra.^23,36^

The Raman map of a fully charged electrode validates the λ-MnO_2_-type structure as its main phase.^23,37^ The main line (A_1g_) is located at 588 cm^–1^ followed by the small peaks at 495 cm^–1^ (F_2g_) and 460 cm^–1^ (E_g_), as well as another minor line (F_2g_) at 640 cm^–1^. It is also worth mentioning that over map investigations, a very small amount (below 1%) of Mn_3_O_4_ compounds are also detected.^38^ Based on a prior analysis of the powder LiMn_2_O_4_ material, it is known that the impurity phases are postsynthetic undesirable residues and can be a partial reason for the initial electrochemical capacity value lower than theoretically expected for the LMO material.

**Figure 4:**
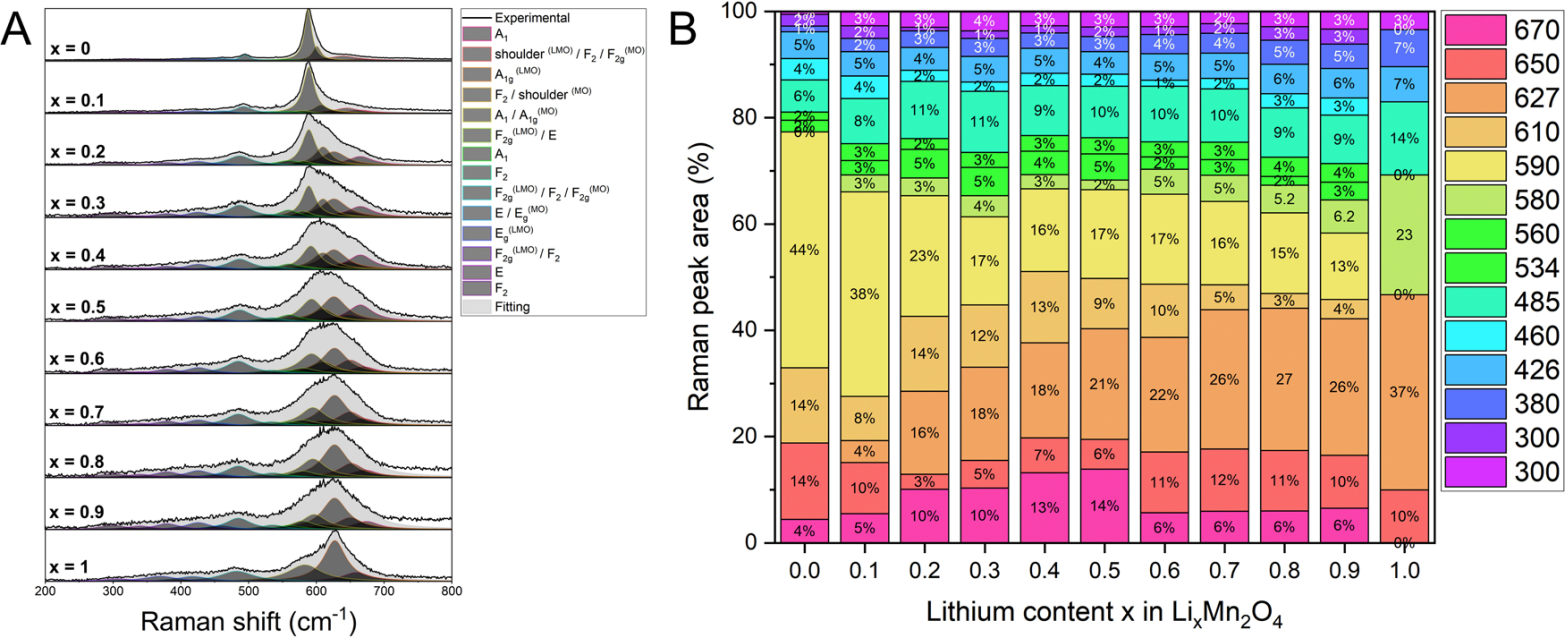
(A) Fitting of the Raman spectra and (B) Raman peak area dependence on delithiation of the Li_*x*_Mn_2_O_4_ structure, where *x* is the Li content (0 ≤ *x* ≤ 1), LMO-LiMn_2_O_4_ and MO-λ-MnO_2_.

The intermediate processes are more varied. At 4.01 V, spectra change and bands from 670 up to 580 cm^–1^ start to rise (Figure 3—blue area and Figure 4). This structural change continues until reaching an intermediate state (4.05 V) where the bands above 600 cm^–1^ decrease, while the band at ca. 592 cm^–1^ takes the lead and becomes the most intense peak in the spectra representing a structure close to Li_0.5_Mn_2_O_4_ and occupying most of the electrode area (green spots). The Li_0.5_Mn_2_O_4_ crystal has Li ions in every second tetrahedral site of fully lithiated LiMn_2_O_4_ (Figure 2B). Spectra around the half-charge state, which originate from Li_1–*x*_Mn_2_O_4_ as well as Li_0.5__–*y*_Mn_2_O_4_ compositions (where *x* and *y* are above 0 and lower than 0.5), are also present and visibly vary from Li_0.5_Mn_2_O_4_. Since diverse spectra coexist within partially charged samples, it shows that LMO grains exhibit the Li intercalation gradient during charging, which can be caused by their differences in the crystal size and thus their different levels of delithiation. Further delithiation (at 4.15 V) during the second plateau demonstrates that the Raman spectra continue to evolve into a highly delithiated phase, although it is not yet a pure λ-MnO_2_-type structure. The fully delithiated phase (λ-MnO_2_ structure) is present only at 4.5 V. The delithiation gradient through the charging process significantly shows that the electrochemical reaction inside grains appears at different speeds. The cause of this effect can be explained by the distribution of grain size within the electrode or the agglomerated structure of the material, as shown in the SEM images (Figure 1). Such a situation can cause the environment for the local overcharging (overpotential) of grains and thus successive material degradation over prolonged cycling.

A comprehensive Raman spectra analysis is performed to track line intensity changes (Figures 4 and S3, S4). The selected spectra are normalized to the most intense peak, carefully fitted (Figure 4A), and for a good presentation of the most intense peaks during charging at different delithiation stages, each peak area is displayed as the percentage of the full area under the spectra (Figure 4B). The Raman fitting was performed considering phase transition stages and the possible overlapping of structures over cycling. The irreducible representations of all three structures are denoted by
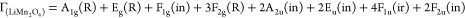

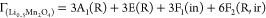

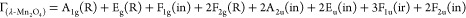
where (R) represents Raman-active vibration, (ir) infrared-active vibrations, and (in) are inactive modes.^23,38^ It means that there are 5 predicted Raman modes in LiMn_2_O_4_, 12 in Li_0.5_Mn_2_O_4_, and 4 in the λ-MnO_2_ structure. These assumptions as well as previous calculations of Raman band positions^18^ in those structures were considered during fitting. All Raman line positions with band assignments and band widths for those selected spectra of Li_*x*_Mn_2_O_4_ across cycling are presented in Table S2.

By such an analysis, the evolution of Raman lines can be tracked. It is visible that the A_1g_ phonon mode (around 627 cm^–1^) of the fully lithiated structure of LiMn_2_O_4_ decreases by about 11% upon the first charging plateau and follows the next intensity decrease within the intermediate *F*4̅3*m* phase formation, whereas it completely vanishes for the highly delithiated λ-MnO_2_ structure. Since 627 cm^–1^ is not present in theoretical Raman modes of Li_0.5_Mn_2_O_4_, the existence of this line is the most probable due to the presence of the solid solution LiMn_2_O_4_–Li_0.5_Mn_2_O_4_ within the grain giving the response from both phases. Upon charging, the F_2g_^(3)^ line (583 cm^–1^) in the lithiated *Fd*3̅*m* structure spits into two lines: at 597 (A_1_) and 580 cm^–1^ (E). During further delithiation, the A_1_ mode of the *F*4̅3*m* phase increases and slightly shifts to 589 cm^–1^ to become a leading peak (A_1g_ phonon mode) for the fully delithiated λ-MnO_2_ structure (588 cm^–1^). The F_2g_^(2)^ line (482 cm^–1^) transforms into the F_2_ line (485 cm^–1^) in the *F*4̅3*m* phase with almost unchanged intensity. After the half-charge state, it changes back into the F_2g_^(2)^ line for the delithiated Fd3̅m structure. The other F_2_ line in the *F*4̅3*m* phase (at 610 cm^–1^) increases over charging and start to decay for lithium concentrations *x* < 0.2. It is also noted that very small traces of lines transformed from the *F*4̅3*m* phase are visible in the Raman fitting of the fully charged sample, and they might be a representation of not-fully charged particles overlapping with the pure λ-MnO_2_ phase even stronger, supporting the Li concentration gradients within the LMO grains at the particular state-of-charge of the electrode.

Figure S3 shows the evolution of spectra from *x* = 0 to *x* = 1 in Li_*x*_Mn_2_O_4_. Horizontal and vertical profiles of the map display the represented Raman spectra at transition stages and normalized intensity changes across charging, respectively. Additionally, an integral intensity ratio between the A_1g_ Raman mode of the lithiated *Fd*3̅*m* structure (ca. 627 cm^–1^) to the sum of the A_1_ mode of the partially delithiated *F*4̅3*m* phase (ca. 592 cm^–1^) and the A_1g_ phonon mode of the highly delithiated λ-MnO_2_ structure (ca. 588 cm^–1^) is shown in Figure S4. It demonstrates that the charge state of the particle can be calibrated and tracked numerically, giving the Raman technique a significant advantage over other structural methods.

### 3.4 Ex Situ Raman Mapping on Electrodes Charged at Different C-Rates

**Figure 5:**
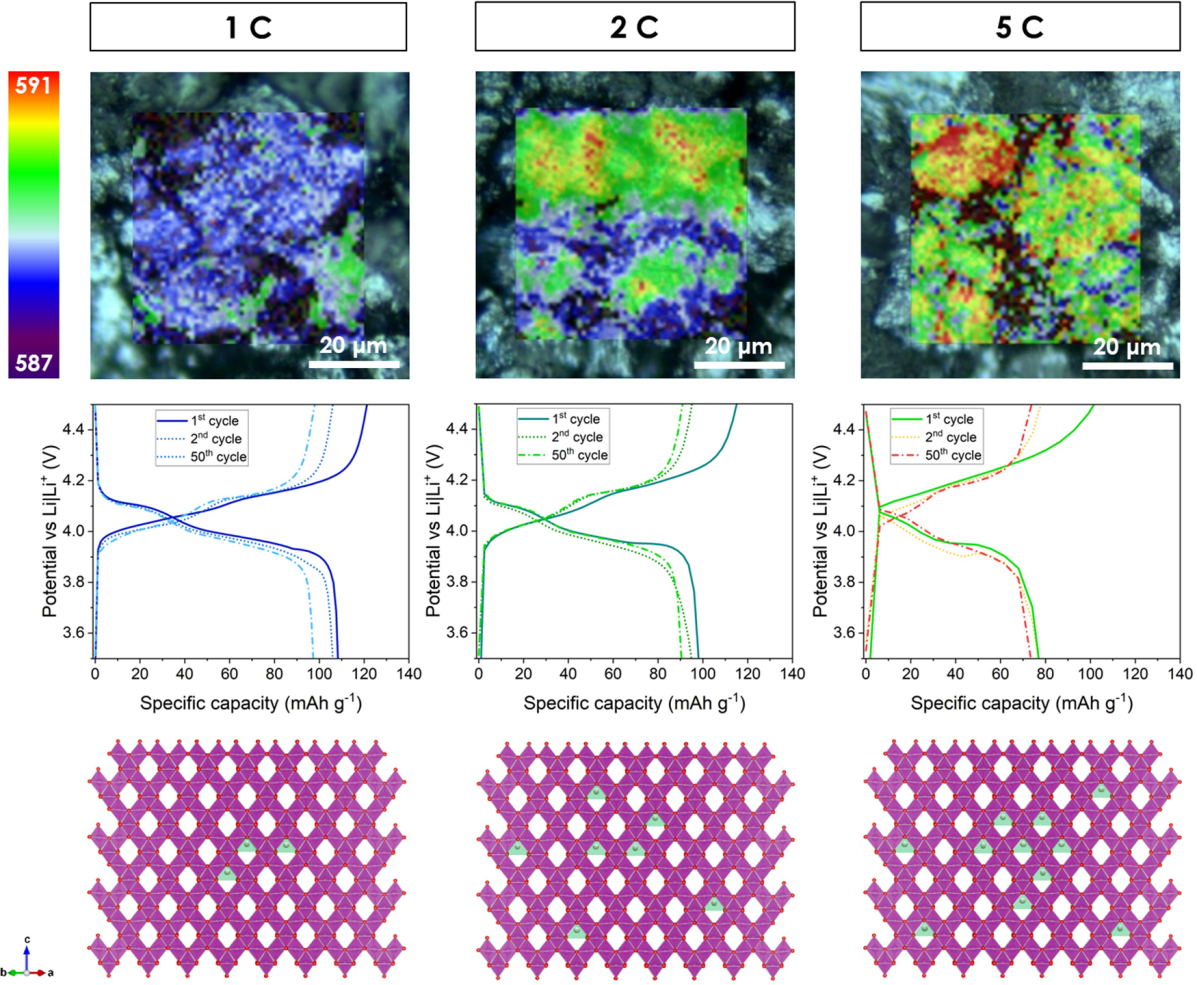
Raman maps of the electrodes charged (up to 4.5 V) after the 50th cycle, charge/discharge curves of the 1st, 2nd, and 50th cycles, and crystal schematics showing the influence of the C-rate from 1 to 5 C on the performance of the Li_1–*x*_Mn_2_O_4_ structure (Li mark in green). Black spots on the map represent conductive carbon.

The developed Raman mapping method was used to study electrodes charged at different C-rates (1, 2, and 5 C). The resulting Raman maps, shown in Figure 5, illustrate the distribution of the highly delithiated structures after 50 cycles. Corresponding spectra are presented in Figure S5. The electrode cycled at a 1 C-rate exhibited the most uniform distribution. The position of the A_1g_ phonon mode of the fully delithiated λ-MnO_2_ structure (violet-blue area) ranges from 587 to 588.5 cm^–1^. Approximately 10% of the map area (green) represents grains of the slightly lithiated *F*4̅3*m* phase structure, where the A_1_ mode position is at ca. 590 cm^–1^. When cycling electrodes at the higher C-rate, the green to red areas indicate the A_1_ mode (position 589–591 cm^–1^) of the *F*4̅3*m* structure rather than the A_1g_ mode of the fully delithiated spinel. At the 2 C-rate, the map shows the areas of the A_1_ mode at 591 cm^–1^. After cycling at the 5 C-rate, it is clear that most of the grains are not fully delithiated, despite using the CCCV method to prepare the samples, where a constant voltage was applied to stabilize the electrode’s potential. The results indicate a limitation in Li^+^ diffusion within the spinel structure and an increase in interfacial resistance at the surface of the spinel crystal after extended cycling at a high rate. This process is visible even at lower C-rates but becomes more pronounced at higher rates. The location of the undercharged areas shows that this effect appears in the middle of the agglomerates. It is less pronounced on the outer sides where the contact with the conductive carbon is higher. This is shown schematically in Figure 5, where Li^+^ ions are still in the middle of the crystal for the 5 C rate charged electrode. Isolated particles with the LMO agglomerates will be highly influenced by this effect due to the electric resistance, lower charge transfer, and inefficient ionic mobility. This shows that the electrical contact of each LMO particle within the electrode layer is crucial for better performance at high-rate applications.

**Figure 6:**
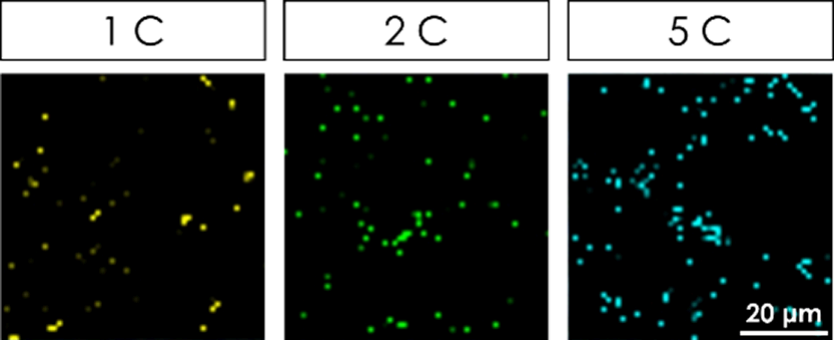
Raman maps of impurities detected at 1, 2, and 5 C. Colored dots represent mixtures of MnO_2_ (todorokite) and Mn_3_O_4_.

Furthermore, we conducted a study on impurity formation, as shown in Figure 6. Cycled electrodes contain minor areas with a combination of additional phases, namely, MnO_2_ (todorokite) and Mn_3_O_4_ (Figure S6).^39^ The concentrations of these mixtures after cycling at 1, 2, and 5 C-rates are ca. 2, 3, and 5%, respectively. These concentrations are higher than the initial concentration of Mn_3_O_4_ detected in the pure electrodes (up to 1%). It is evident that the amount of these phases increases with an increased C-rate. This indicates that the delithiated spinel structure gradually transitions into other Mn_*x*_O_*y*_ phases after extended cycling, and the process is strengthened for higher C-rates. This is in agreement with previous TEM studies, where the transition from the λ-MnO_2_ structure into the Mn_3_O_4_ phase was observed at 4.3 V.^40^ The formation of the monoclinic MnO_2_ (todorokite) might be a result of the Mn^2+^ dissolution from the tetrahedral sides of Mn_3_O_4_ and the creation of the Mn-deficient decomposition product. The coexistence of both reconstruction phases (MnO_2_ todorokite and Mn_3_O_4_) in the same Raman spots strengthens this conclusion. This effect may be due to the Li concentration gradient among LMO particles, resulting from the local under- and overcharging occurring within the same electrode. As impurities are mainly found at the edges of the agglomerates, where particles have good electrical contact with conductive carbon, it is suggested that for higher C-rates, the crystal reconstruction may be caused by a locally higher potential (local overcharge) appearing on the particles located on the outer side of the agglomerate, inducing these structural reconstructions (Figure S7).

## 4 Discussion

Raman microscopy serves as an advantageous instrument for the examination of materials in Li-ion batteries. Its notable sensitivity facilitates in-depth insights into structural differences within the crystals under investigation. Spinel-based electrodes, monitored throughout the charging process, offer a robust model for such investigations. Raman mapping allows for the exploration of phase concentration changes during cycling, aiding in the assessment of potential factors influencing the electrochemical performance of the material. This technique presents a distinctive opportunity to gain a more comprehensive understanding of the underlying mechanisms governing reactions and capacity degradation and provides answers that are challenging to ascertain through electrochemical measurements alone or structural methods that use bulk-type measurements.

When compared to other experimental techniques, Raman microscopy is widely employed in laboratories for several compelling reasons. It is cost-effective, highly sensitive, and capable of detecting even subtle structural changes and offers precise and statistically significant results as the Raman mapping area of the investigated electrode expands. For instance, the conventional X-ray diffraction (XRD) method is insufficiently sensitive for gathering such data, mainly due to bulk-type measurements and the need for extended data acquisition times, resulting in a lack of specificity when compared to Raman spectra. This limitation arises from the small quantity of active material applied in a laboratory-scale electrode, typically in the range of 2–5 mg for 10 mm electrodes, rendering standard characterization methods inadequate, as they fall far below the required detection limits. Raman mapping offers the unique ability to analyze electrode surfaces for their crystallographic homogeneity including the possible detection of impurity phases resulting from synthesis or electrochemical side reactions. It can be complemented by an in-depth investigation of the powder material to prevent the unwarranted overinterpretation of electrochemical data.

In the context of the LMO material, Raman results unmistakably reveal an electrochemical phase transition reaction. The progression of peaks and their subtle shifts serve as precise indicators of the battery’s state-of-charge (SoC) and the coexistence of both Li-rich and Li-poor regions within partially charged electrodes. This underscores that Raman mapping stands as a more precise method for investigating the electrode’s SoC when compared to conventional single-spot Raman measurements.

The lithiation gradient across the extensive surface area provides a distinctive perspective of the electrochemical reaction mechanism. The Li concentration gradient within the crystal was also observed during in situ TEM studies of the LMO nanowire.^41^ During rapid charge and discharge, the nanowire exhibited distinct Li-rich and Li-poor phases separated by a transition region. This transition region reversibly moved along the nanowire to facilitate the transport of lithium ions. Our detected spectral gradient in the Raman maps of the partially lithiated LMO material additionally confirms the above-mentioned mechanism. Following this finding, we employed Raman mapping to investigate electrodes charged at different C-rates (1, 2, and 5 C) over 50 cycles, revealing distinct distributions of delithiated structures. Notably, the Raman maps varied significantly depending on the C-rate, with the 1 C-rate showing the most uniform distribution of the fully delithiated phase. At higher C-rates (2 and 5 C), the maps showed increased areas of the slightly lithiated *F*4̅3*m* structure, indicating limitations in Li^+^ diffusion within the spinel structure and increased interfacial resistance.

Furthermore, Raman mapping can be used as a very sensitive tool for the detection of impurity formation detection. We were able to observe additional phases including MnO_2_ (todorokite) and Mn_3_O_4_ structures within cycled electrodes, with their concentrations increasing with higher C-rates. This suggests potential crystal reconstruction due to a locally higher potential (local overcharging) and underscores the importance of optimizing electrical contact within LMO agglomerates for enhanced performance in high-rate applications.

## 5 Conclusions

Examining the electrode surface by Raman mapping provides the opportunity to monitor the lithiation state of particles at specific potentials vs Li. The evidence of the phase transition between LiMn_2_O_4_ into λ-MnO_2_ with a significant contribution of the Li_0.5_Mn_2_O_4_ intermediate state is observed. Furthermore, mechanisms complementing electrochemical processes and comprehensive assignments of each detected phase are performed. In this study, we monitor changes in the *F*4̅3*m* phase for all stages for lithium concentration higher than 0 and lower than 1. Raman mapping reveals a gradient distribution of Li concentration within the particles at particular potentials, as indicated by variations in the Raman response within the same electrode. In addition, samples operated at high C-rates for 50 cycles were evaluated for their rapid delithiation capabilities and to track impurity formation over harsh cycling conditions. Remarkably, even small traces of impurity phases, typically below the detection limit of X-ray diffraction, can be detected. Analysis of differences in Raman peak intensities between particles can provide an analytical method to track structural changes and phase gradients within the electrode. This method underscores the notable influence of particle/crystal size on irregularities in the lithiation state and possible local overcharging causing material degradation.

Due to its high sensitivity, spatial resolution, simplicity in sample preparation, and relatively short detection times, Raman spectroscopy proves to be an effective method for the exploration of Li-ion battery electrodes. Its utility extends beyond as-synthesized materials, thus enabling insights into structural changes, phase distribution, and impurities such as byproducts resulting from side reactions within the electrode layer. Ex situ Raman mapping of electrodes is a valuable tool for compositional analysis. It allows for the evaluation of the phase heterogeneity during cycling and provides an approximation of impurities and inactive component phase concentrations. This approach can readily find applications in industrial settings, such as investigating the reasons for capacity fading and determining electrode compositions. Among ex situ experimental techniques employed in lithium-ion battery research, Raman mapping is a suitable tool for comprehensive system analysis thanks to good resolution capabilities and the ability to provide statistically significant data. In this research, we highlight the wide-ranging potential of Raman mapping as a valuable analytical tool for structural and compositional determination, both in research and industrial applications.

## Supporting Information Available

Detailed information about literature data on LiMn_2_O_4_ using ex situ and in situ Raman studies; Raman positions and band widths for selected spectra across Li_*x*_Mn_2_O_4_ cycling; electrochemical results of (A) specific capacity and (B) relative capacity over cycling of LiMn_2_O_4_; Raman spectra of the fully discharged sample showing the shift in A_1g_ line from 627 to 635 cm^–1^; spectral map showing changes within delithiation of the Li_*x*_Mn_2_O_4_ material including profiles within *X-* and *Y*-axis; peak height ratio between the A_1g_ Raman mode of the lithiated Fd3̅m structure and sum of T_2g_(3) of the same structure, A_1_ mode at 590 cm^–1^ from the *F*4̅3*m* phase, and A_1g_ phonon mode of the highly delithiated λ-MnO_2_ structure; Raman spectra from colored map areas (Figure 5) representing the position of the A_1g_ and A_1_ modes of the fully delithiated λ-MnO_2_ structure and the slightly lithiated *F*4̅3*m* structure, respectively; examples of the Raman spectra showing MnO_2_ (todorokite) and Mn_3_O_4_ compounds detected in the electrodes cycled at 1, 2, and 5 C; and crystal structure comparison between MnO_2_ (todorokite), Mn_3_O_4_, and λ-MnO_2_ (PDF)
