# Supplementary material for: Ex Situ Raman Mapping of LiMn2O4 Electrodes Cycled in Lithium-Ion Batteries
Source: ACS Omega. 2024 Jul 1;9(28):30381–91. doi: 10.1021/acsomega.4c01480 (PMC11256290; doi:10.1021/acsomega.4c01480)
Supplement: Supplementary file 1 — ao4c01480_si_001.pdf [file ao4c01480_si_001.pdf]

Ex-situ Raman mapping of $LiMn_2O_4$ electrodes cycled in lithium-ion batteries

Dominika A. Buchberger[1,], Bartosz Hamankiewicz[1], Monika Michalska[2], Alicja Głaszczka[1], Andrzej Czerwinski[1]*

[1] Faculty of Chemistry, University of Warsaw, Pasteura 1, 02093 Warsaw, Poland

[2] Faculty of Materials Science and Technology, VSB-Technical University of Ostrava, 17. listopadu 2172/15, 708 00, Ostrava-Poruba, Czech Republic

Corresponding author: d.buchberger@uw.edu.pl

KEYWORDS

ex-situ Raman, Raman mapping, electrode materials, spinel $LiMn_2O_4$, Li-ion batteries

Supporting Information

Supporting material – table of contents

Table S1. Literature data on $LiMn_2O_4$ using ex situ and in situ Raman studies. **S-3**

Table S2. Raman line positions and band widths for selected spectra across $Li_XMn_2O_4$ cycling. **S-4**

Figure S1 Electrochemical results of (A) specific capacity and (B) relative capacity over cycling of $LiMn_2O_4$. **S-5**

Figure S2 Raman spectra of fully discharged sample showing the shift in A_{1g} line from 627 to 635 cm^{-1}. The inset represents corresponding Raman map of the A_{1g} line position. **S-6**

Figure S3. Spectral map showing changes within delithiation of $LixMn_2O_4$ material including profiles within X and Y axis. **S-7**

Figure S4. Integral intensity ratio between A_{1g} Raman mode of lithiated $Fd\overline{3}m$ structure and the sum of A_1 mode at 590 cm^{-1} from $F\overline{4}3m$ phase, and A_{1g} phonon mode of highly delithiated λ-MnO_2 structure. **S-8**

Figure S5. Raman spectra from colored map areas (Figure 5) representing the position of the A_{1g} and A_1 modes of the fully-delithiated λ-MnO_2 structure and the slightly lithiated $F\overline{4}3m$ structure, respectively. **S-9**

Figure S6. Examples of the Raman spectra showing MnO_2 (todorokite) and Mn_3O_4 compounds detected in the electrodes cycled at 1C, 2C and 5C. **S-10**

Figure S7. The crystal structure comparison between MnO_2 (todorokite), Mn_3O_4 and λ-MnO_2. **S-11**

Table S1. Literature data on LiMn2O4 using ex situ and in situ Raman studies.

Ref.	Year	Authors	Raman measurement type	Novelty	Electrochemical cell	Working electrode	Electrolyte	Reference and counter electrodes	Raman system	Laser used (power)
17	1998	Kanon et al.	**in situ** & ex situ	The first in situ Raman study of electrochemical Li insertion in MnO2 spinel structure in aqueous solution.	three-electrode: simple aqueous electrochemical cell	*in situ* : thin layer electrode Pt/λ-MnO2	0.1 M LiCl + 0.05 M borate buffer aqueous solution (pH 7.5)	Ref: Ag/AgCl electrode Count.: Pt plate	HoloProbe 532 system (Kaiser Optical Systems, Co., Ltd.) with microscope (immersed objective)	Nd:YAG laser at 532 nm (power output: 1mW)
18	1999	Ammundsen et al.	ex situ	Theoretical calculation of Raman active phonon positions in LiMn2O4, Li0.5Mn2O4 and MnO2 and experimantal evidence.	two-electrode: simple aqueous electrochemical cell	*ex situ* : thin film electrode Pt/LMO & further Pt/λ-MnO2	0.01 M LiCl aqueous solution	Ref.: saturated calomel electrode	Holoprobe 532 Raman system (Kaiser Optical Systems Inc.) with a microscope attachment	Nd:YAG laser at 532 nm (power: unknown)
19	1999	W. Huang & R. Frech	**in situ**	First in situ Raman studies of LMO electrodes during lithium intercalation in Ar sealed Li-ion cell cycled between 4.6 and 2.1 V vs. Li metal	two-electrode: split type cell (sealed under Ar)	*In situ* : electrode films - 85 wt.% AM, 10 wt.% Super-P, 5 wt.% PVdF in cyclopentanone onto Al meshes.	1 M LiClO4 in 1:1 wt. EC:DMC (Celgard 2400 separator)	Ref./Count.: Li metal	JobinYvon ISA T64000 Raman spectrometer with a microscope	Ar laser: 514.5 nm line (power input: 20 mW)
20	2001	Y. Luo et al..	**in situ**	This research exploits the capabilities of in situ Raman technique for the acquisition of time-resolved Raman spectra of single particles of LiMn2O4 embedded in Au foil substrate electrodes as a function of the applied potential.	two-electrode: split type cell (sealed under Ar)	*in situ* : LiMn2O4 particle electrodes in Au foil	1 M LiPF6 in 2:1 wt. EC/DMC	Ref./Count.: Li metal	Raman 2000 system (Chromex Inc., Albuquerque, NM) with a microscope attachment	Nd:YAG laser at 532 nm (power: ~8mW)
21	2003	K. Dokko et al..	**in situ**	Statistical analyses of the spectra in the range 15% < SOD < 45% showed to be consistent with the coexistence of two distinct phases of lithiated metal oxide and agreeed weell in situ XRD measurements.	two-electrode: microelectrode self-design cell (sealed under Ar)	*in situ* : the microelectrode Pt/ LiMn2O4 microparticle	1 M LiClO4 1:1 vol. EC:DEC	Ref./Count.: Li metal	Raman 2000 system (Chromex Inc., Albuquerque, NM) with a microscope attachment	Nd:YAG laser at 532 nm (power: low)
22	2003	N. Anzue et al.	**in situ** & ex situ	The first to report in situ Raman measurements of structural changes of an electrostatic spray deposited thin-film (without binder or conductive agents) of Li1-xMn2O4 during lithium insertion and extraction processes.	two-electrode: microelectrode self-design cell (sealed under Ar)	*in situ* : Li1 - xMn2O4 thin films produced by electrostatic spray deposition (no binder, no conductive carbon)	1 M LiClO$_4$ in PC/EC	Ref: Li foil Count.: Li foil	Raman spectrometer (self-assembled)	Ar laser: 514.5 nm line (power: unknown)
23	2003	C.M. Julien & M. Massot	ex situ	The study of local structure of various lithium manganese oxides stoichiometries using both the classical group factor analysis and a local environment model.	unknown (electrochemical Li extraction)	ex situ: unknown (Li0.5Mn2O4 sample)	unknown	unknown	Jobin-Yvon U1000 laser Raman spectrometer	Ar laser: 514.5 nm line (power density: 10 Wcm^{-2})
24	2005	Q. Shi et al.	**in situ**	The contributions of the three crystallographic phases of LixMn2O4 0 < x < 1 as a function of the amount of Li+ in the lattice derived from the optical data were consistent with those extracted from a coulometric analysis of the voltammetric curves.	two-electrode: microelectrode self-design cell (sealed under Ar)	*in situ* : LiMn2O4 single crystal microelectrode	1 M LiPF6 in 1:1 vol. EC/DMC	Ref./Count.: Li metal	Raman 2000 system (Chromex Inc., Albuquerque, NM) with a microscope attachment	Nd:YAG laser at 532 nm (power: 3 mW)
25	2015	H.-Y. Amanieu et al.	ex situ	*Ex situ* Raman study on the commercial LMO electrodes at their charged and discharged states.	two-electrode: standard commercial cells with graphite	ex situ: LMO electrode from commercial cell	unknown	graphite	confocal LabRAM Aramis Raman spectrometer (Horiba Jobin Yvon)	unknown
26	2017	N. Leifer et al.		The correlation between the electrochemical data and structural responses of LMO electrodes (commercial LMO material) charged to high anodic potentials of 4.3 to 5.1 V vs. Li	two-electrode: pouch-type cells with Li foil as counter electrodes and Celgard polypropylene separator	in situ: LiMn2O4, CB, and PVdF (80:10:10 by weight) on Al foil (~20 μm thick)	1 M LiPF6. in 7:3 wt. EMC/EC	Ref./Count.: Li metal	Renishaw inVia micro-Raman spectrometer	514 nm laser (power output: ~0.23 mW)
27	2018	B. Slautin et al..	ex situ	The understanding of delithiation and degradation paths by local CRM measurements of LMO cathode material with different 'state of charge' and 'state of health' parameters.	two-electrode: commercial cylindrical cells	ex situ: LMO electrode from commercial cylindrical cells	unknown	unknown	confocal Raman microscope (Alpha 300 AR, WiTec GmbH)	488 nm laser (about 260 nm spatial resolution)

Table S2 Raman line positions and band widths for selected spectra across $Li_XMn_2O_4$ cycling.

Raman mode & width	Phase	Li concentration in $Li_xMn_2O_4$										
		1	0.9	0.8	0.7	0.6	0.5	0.4	0.3	0.2	0.1	0
A_1	$Li_{0.5}Mn_2O_4$		674	672	670	670	665	665	665	665	665	
w			45	45	45	45	45	45	45	45	45	
shoulder	$LiMn_2O_4$	659										
w		50										
F_2 / F_{2g}	$Li_{0.5}Mn_2O_4$ / λ-MnO_2		650	652	650	650	647	647	647	647	645	640
w			45	45	45	45	45	45	45	45	45	50
A_{1g}	$LiMn_2O_4$	627	627	627	627	627	626	626	626	626	626	
w		40	45	45	45	45	45	45	45	45	45	
F_2 / shoulder (MO)	$Li_{0.5}Mn_2O_4$		610	611	610	610	610	610	610	610	607	
w			45	45	40	40	40	35	30	30	25	
shoulder	λ-MnO2											601
w												15
A_1 / A_{1g}	$Li_{0.5}Mn_2O_4$ / λ-MnO_2		597	595	595	593	593	592	591	590	589	588
w			45	45	40	40	33	30	25	25	20	13
F_{2g} / E	$LiMn_2O_4$ / $Li_{0.5}Mn_2O_4$	583	580	580	580	580	580	580	580	580	579	
w		60	45	45	40	40	40	35	30	30	30	
A_1	$Li_{0.5}Mn_2O_4$		560	560	560	560	560	560	559	559	559	
w			45	45	45	40	40	45	35	45	35	
F_2	$Li_{0.5}Mn_2O_4$		536	534	534	534	534	534	532	532	532	
w			45	45	45	45	45	45	45	45	45	
F_{2g} / F_2 / F_{2g}	$LiMn_2O_4$ / $Li_{0.5}Mn_2O_4$ / λ-MnO_2	482	485	485	485	487	487	487	487	487	493	495
w		60	45	45	45	45	45	45	45	45	30	17
E / E_g	$Li_{0.5}Mn_2O_4$ / λ-MnO2		460	460	460	460	460	460	460	460	460	460
w			45	45	45	45	45	45	45	45	45	30
E_g	$LiMn_2O_4$	418	426	426	426	426	426	426	426	426	420	
w		60	45	45	45	45	45	45	45	45	45	
F_{2g} / F_2	$LiMn_2O_4$ / $Li_{0.5}Mn_2O_4$	370	380	380	380	380	380	380	380	380	380	
w		60	45	45	45	45	45	45	45	45	45	
E	$Li_{0.5}Mn_2O_4$		340	340	340	340	340	340	340	340	335	
w			45	45	45	45	45	45	45	45	45	
unknown / F_2	$LiMn_2O_4$ / $Li_{0.5}Mn_2O_4$	306	300	300	300	300	300	300	300	300	300	
w		60	45	45	45	45	45	45	45	45	45	

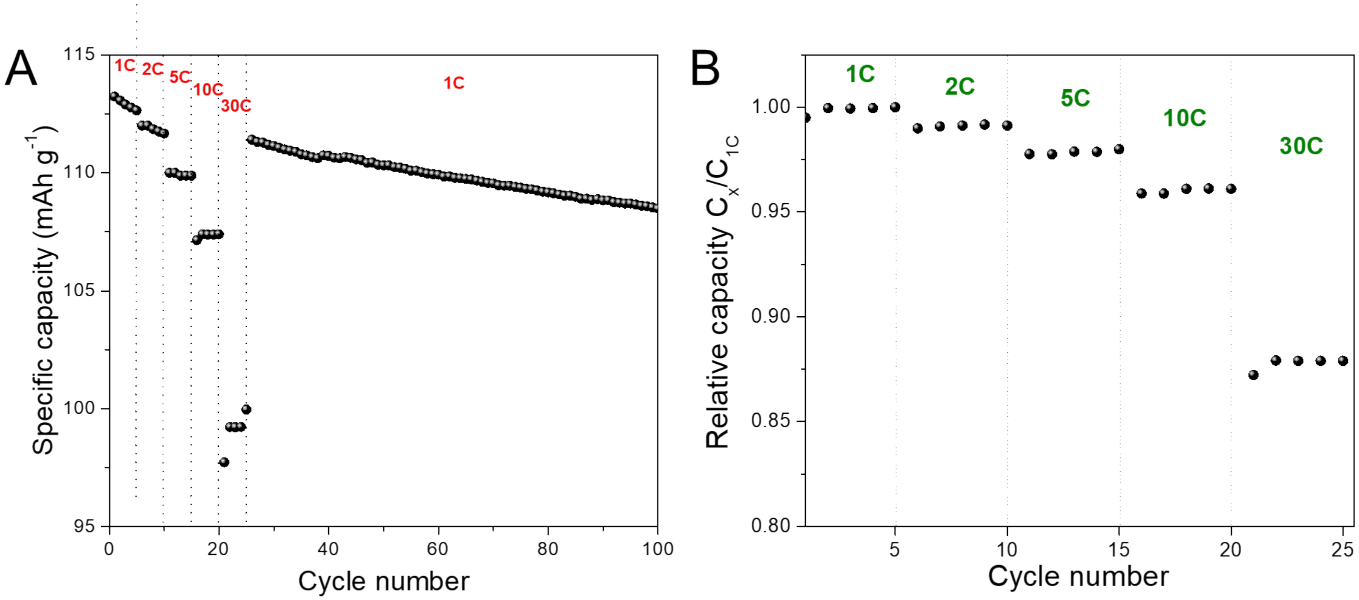

Figure S1. Electrochemical results of (A) specific capacity and (B) relative capacity over cycling of $LiMn_2O_4$.

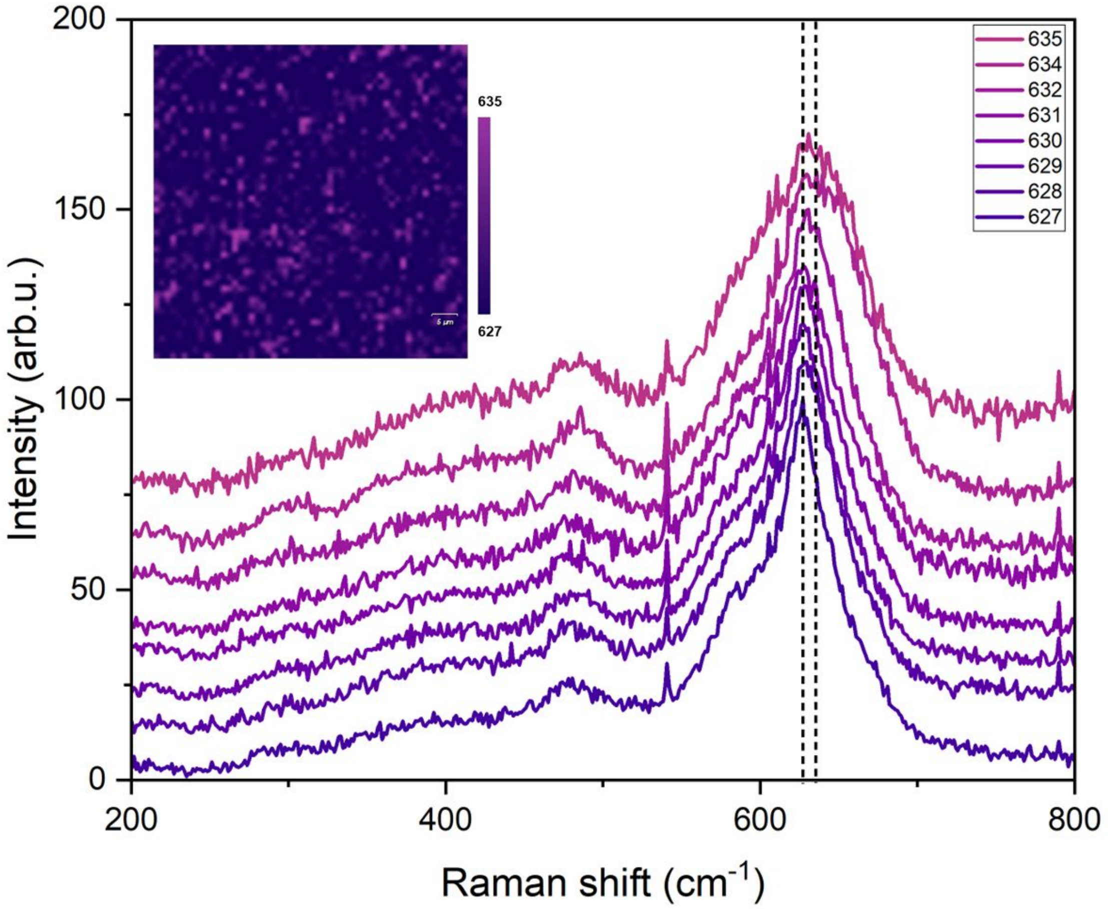

Figure S2. Raman spectra of fully discharged sample showing the shift in A_{1g} line from 627 to 635 cm^{-1}. The inset represents corresponding Raman map of the A_{1g} line position.

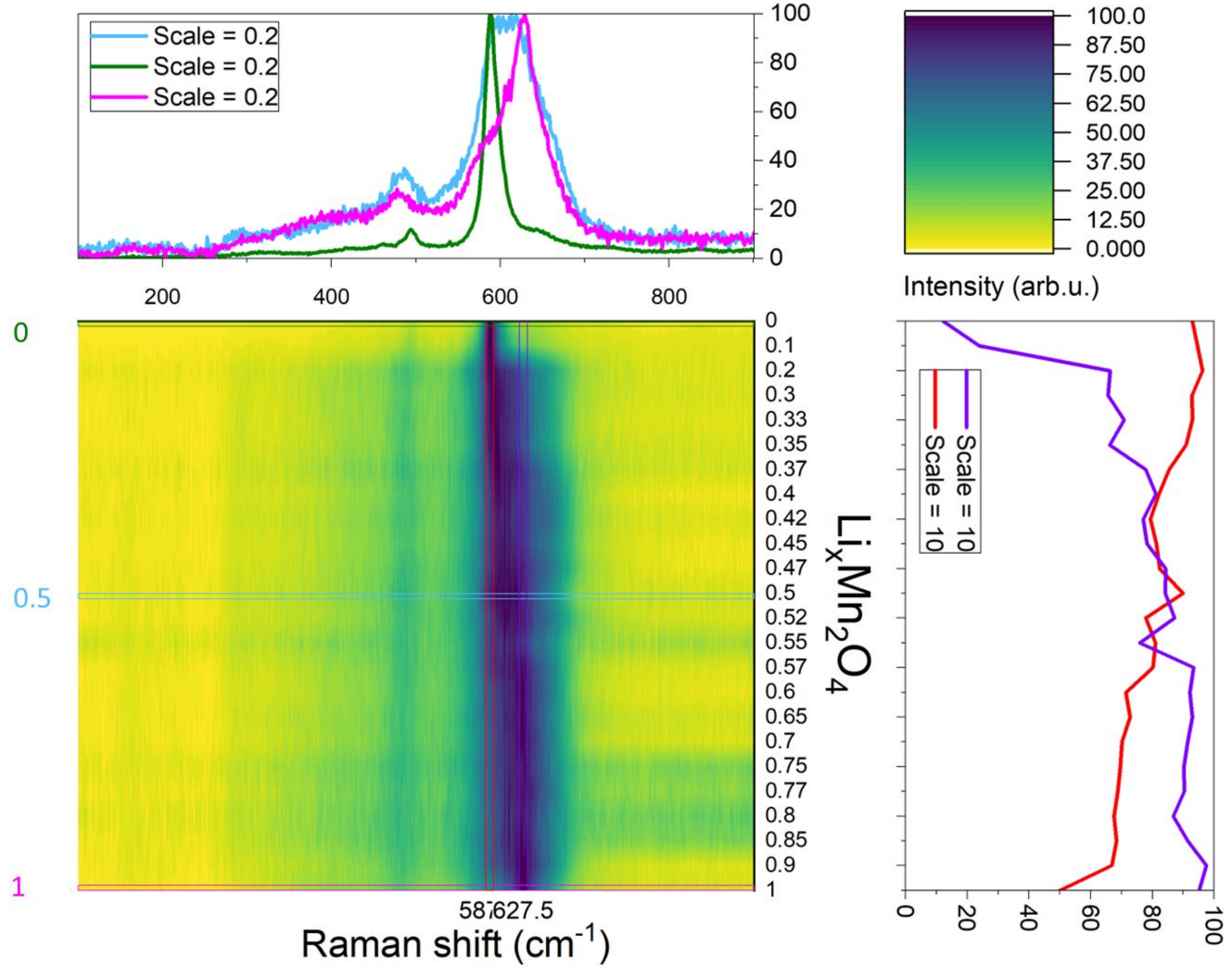

Figure S3. Spectral map showing changes within delithiation of LixMn$_2$O$_4$ material including profiles within X and Y axis.

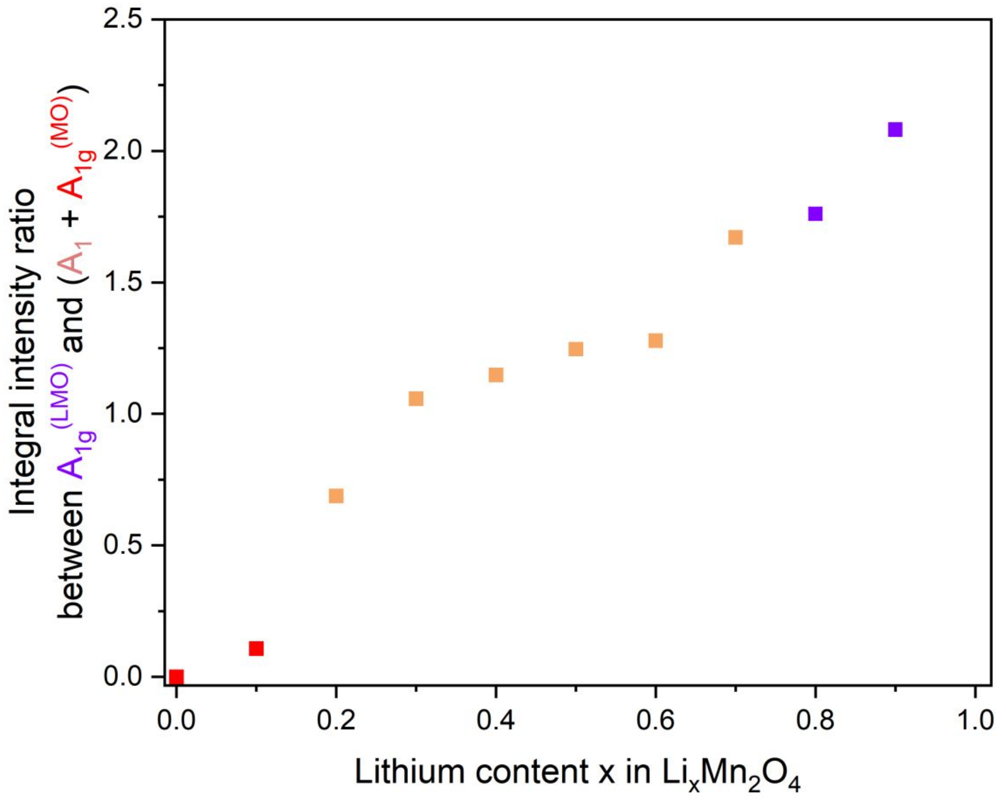

Figure S4. Integral intensity ratio between A_{1g} Raman mode of lithiated Fd$\bar{3}$m structure and the sum of A_1 mode at 590 cm^{-1} from F$\bar{4}$3m phase, and A_{1g} phonon mode of highly delithiated λ-MnO_2 structure.

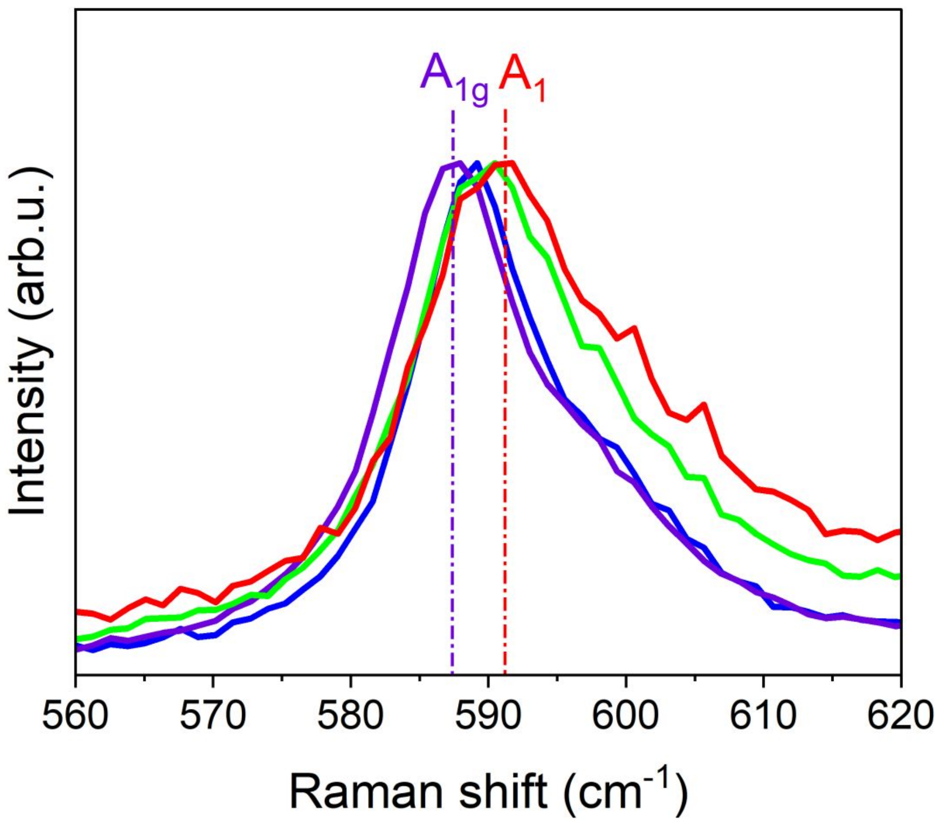

Figure S5. Raman spectra from colored map areas (Figure 5) representing the position of the A_{1g} and A_1 modes of the fully-delithiated λ-MnO_2 structure and the slightly lithiated $F\overline{4}3m$ structure, respectively.

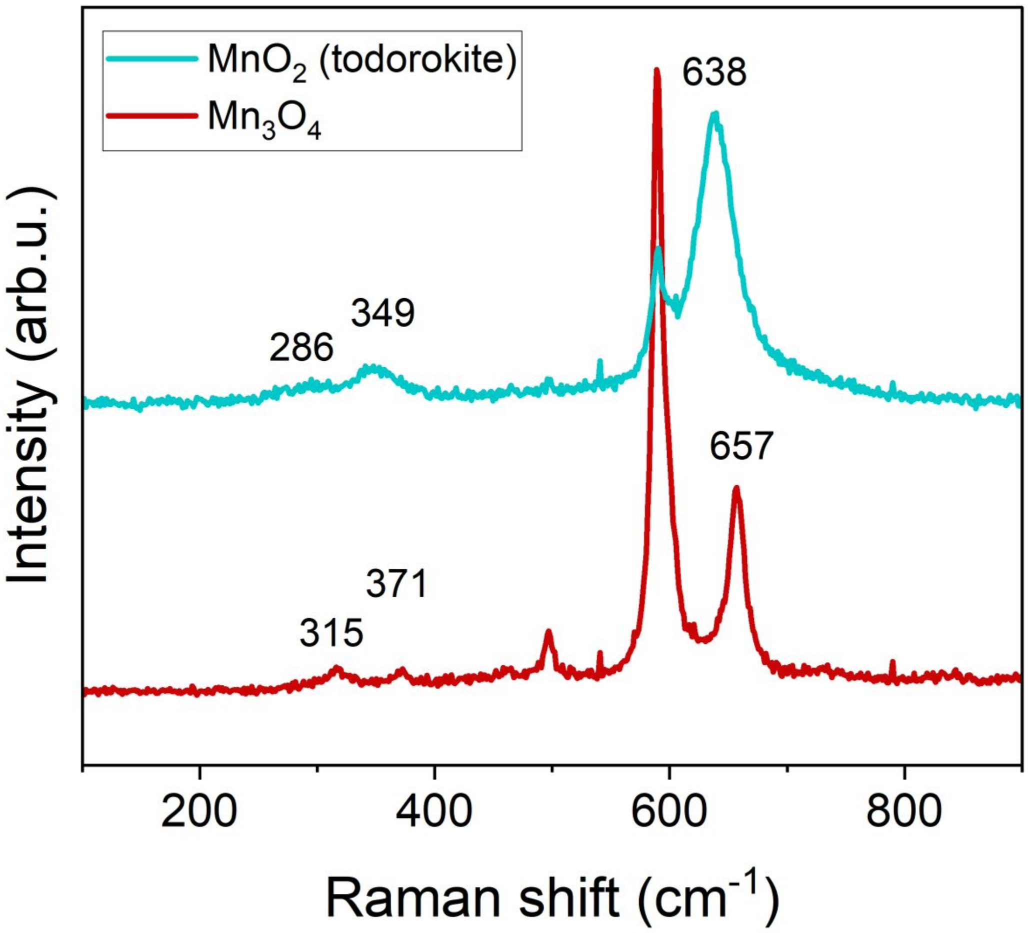

Figure S6. Examples of the Raman spectra showing MnO_2 (todorokite) and Mn_3O_4 compounds detected in the electrodes cycled at 1C, 2C and 5C.

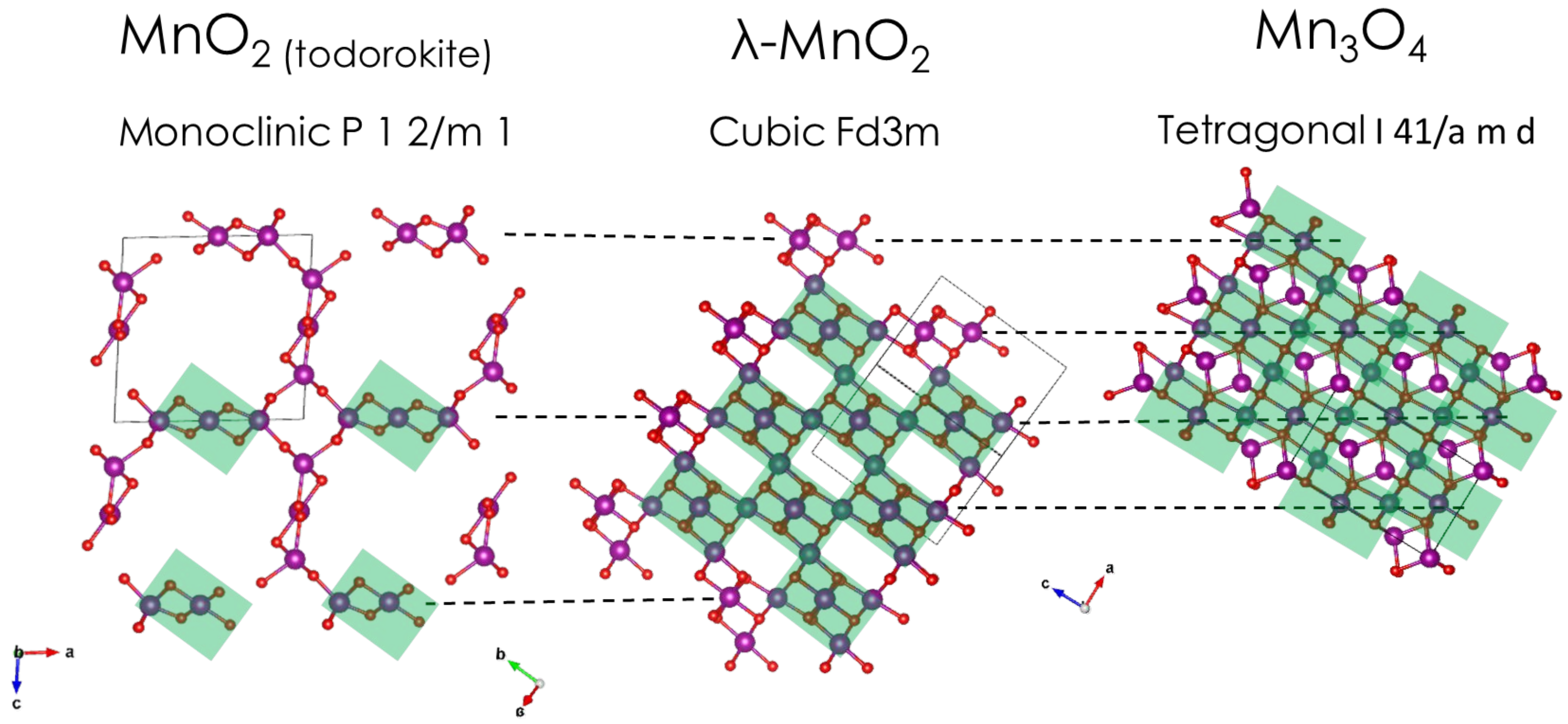

Figure S7. The crystal structure comparison between MnO_2 (todorokite), Mn_3O_4 and λ-MnO_2.
